# Multimodal Data Fusion for a Self-Adaptive, Smart, Serious-Game Ecosystem Under Development

**DOI:** 10.3390/s26165132

**Published:** 2026-08-13

**Authors:** Xiya Tao, Peng Chen, Martina Eckert

**Affiliations:** Escuela de Ingeniería y Sistemas de Telecomunicación (ETSIST), Centro de Electrónica Industrial y Sistemas Multimodales (CEIMM), Universidad Politécnica de Madrid (UPM), Campus Sur, Calle Alan Turing s/n, 28031 Madrid, Spainmartina.eckert@upm.es (M.E.)

**Keywords:** multimodal data fusion, serious games, rehabilitation, feature-level fusion, user-state estimation

## Abstract

This article presents the implementation and technical feasibility evaluation of the multimodal sensing and feature-level fusion layer of BLEXER v3, a broader serious-game ecosystem under development for upper-limb rehabilitation. The implemented framework integrates Kinect-based motion tracking, Polar H10 and Bangle.js physiological sensing, wearable accelerometer data, and facial affective cues within a middleware-based architecture. Heterogeneous sensor streams are locally preprocessed, temporally aligned, and transformed into a common quality-aware multimodal feature representation containing motion, heart-rate and heart-rate-variability-related descriptors, affective information, availability indicators, signal-quality metadata, and freshness descriptors. The fused representation is additionally mapped, using predefined rules, to heuristic operational descriptors, including low demand, moderate stable, active engagement, physical load, affective activation, high demand, and uncertain. These descriptors are not intended as clinical diagnoses, independently validated user states, or final adaptation decisions. An exploratory K-means analysis of 12,675 complete multimodal windows reveals partial correspondence between the data-driven cluster structure and the predefined operational descriptors. Some descriptors show comparatively concentrated cluster patterns, whereas others exhibit overlap and internal heterogeneity. The results demonstrate the technical feasibility of generating structured multimodal representations that can provide input for subsequent context-aware reasoning. Independent validation of the operational descriptors, completion and evaluation of the whole system, and clinical validation with rehabilitation patients remain future work.

## 1. Introduction

The development of human–computer interaction (HCI) has increasingly relied on intelligent sensing technologies that can perceive, interpret, and respond to human behaviour in real time. In adaptive interactive systems, the integration of physical, physiological, and emotional signals enables context-sensitive responses that are more personalised, responsive, and effective. This capability is particularly relevant in rehabilitation scenarios, where the user’s condition may vary continuously during training and where timely adaptation is essential to maintain both safety and engagement.

To overcome these limitations, our research group is developing the BLEXER system (Biosignal and Learning-based Exercise Regulation), a complex ecosystem designed for personalised physical exercises using serious games. Earlier versions of BLEXER focused exclusively on motion-based game control and manual parameter adjustment to be done by a therapist [1,2]; the latest generation, which is currently under development, BLEXER v3 [3], will be based on a modular architecture aimed at supporting and assisting the therapist by making automated decisions on difficulty adjustment. The most recently created module for that architecture, the Sensor Module (SM), is the centre of the work presented here: inside the module, different types of data obtained from the user during play are preprocessed through multimodal data fusion to provide a structured multimodal representation of the current interaction condition.

The integration of heterogeneous sensor signals into a coherent and operational representation of the user state is a challenge: motion, physiological, and affective data differ in sampling rate, temporal stability, semantic meaning, and susceptibility to noise, which makes direct combination difficult in real-time rehabilitation scenarios. Therefore, subsequent context-aware reasoning and adaptation require not only multimodal sensing but also a fusion strategy capable of preserving modality-specific information while producing a structured representation for downstream processing.

Existing rehabilitation-oriented sensing approaches often focus either on individual sensing modalities or on complete user-state recognition and adaptive decision-making systems. However, reliable state estimation and adaptation require an intermediate sensing and feature-level fusion layer that can organise heterogeneous data before higher-level reasoning is attempted. The present work focuses specifically on that intermediate layer.

The objective of this work is to implement and technically evaluate a multimodal sensing and feature-level fusion pipeline capable of synchronising heterogeneous behavioural, physiological, and affective information and generating a structured representation suitable for subsequent context-aware reasoning. A secondary exploratory objective is to examine whether the resulting fused multimodal feature space contains recurring data-driven structures and to assess their correspondence with the predefined rule-based operational descriptors.

The main contributions of this work are fourfold: (1) an implemented multimodal sensing framework that integrates motion, physiological, wearable, and affective sensing channels in a rehabilitation-oriented serious-game setting; (2) a feature-level fusion pipeline that performs window-level feature extraction, temporal alignment, availability annotation, and quality annotation; (3) a rule-based operational representation that summarises multimodal interaction patterns as a technical basis for future context-aware reasoning; and (4) an exploratory unsupervised clustering analysis examining the degree of correspondence between recurring data-driven structures and the predefined operational descriptors.

## 2. Materials and Methods

**Figure 1:**
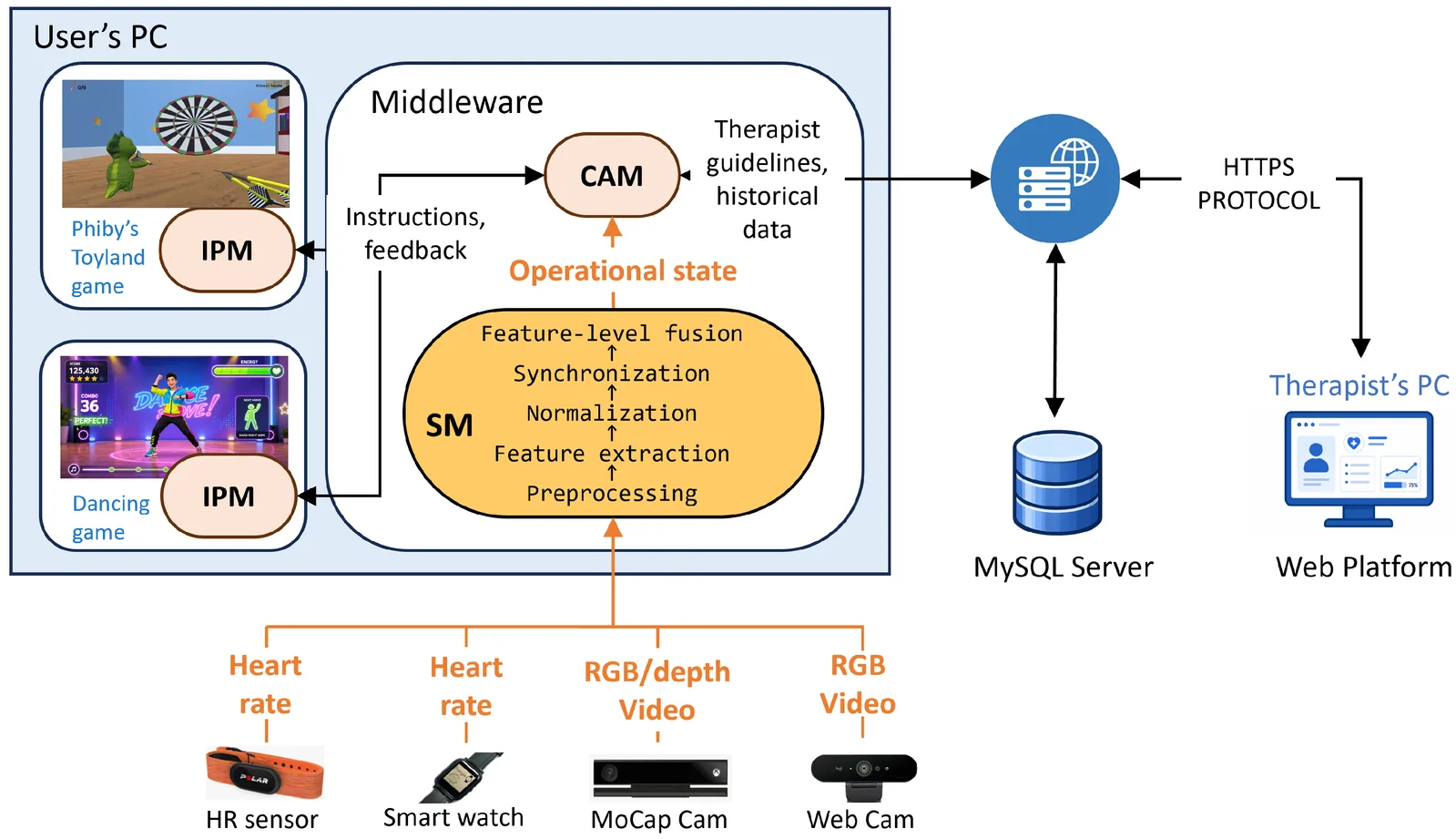
Overview of the BLEXER v3 ecosystem architecture and its main modules.

To clarify the role of the multimodal data fusion component developed in this work, Section 2.1 firstly presents the global architecture of the BLEXER v3 environment (Figure 1), introducing the main functionalities of the modules (orange blocks) that will be added to transform the previous system into a self-adaptive, smart, serious-game ecosystem. In Section 2.3, the focus is set on the Sensor Module, which houses the contributions of this work.

### 2.1. BLEXER v3 Ecosystem Architecture

The architecture is built on the previous development stages of the BLEXER platform [1,2,4] with three main components: one or more serious games, a web server with a database for personalised game difficulty adjustment and monitoring of the patient’s progress, and middleware that transmits data between the MoCap sensor, games, and database. In this way, the adaptation was bound to therapist-defined adjustments; no automatic control was provided. Figure 1 visualises how BLEXER v3 extends this foundation by introducing a modular architecture designed to enable automated, intelligent decisions wherever possible. These decisions are made by the Context-Awareness Module (CAM), which is integrated into the middleware and therefore needs to be based on context knowledge such as rehabilitation objectives (provided by the therapist), current gaming performance and evolution, as well as the physiological and affective state of the user. While game-related data can be provided directly by the game (via the Intelligent Play Module (IPM)), physiological and affective data must be captured by various sensors and are therefore of different types, e.g., motion data are obtained by a depth camera (Kinect (Microsoft Corporation, Redmond, WA, USA)), heart rate is obtained by a smartwatch or a breastband, and emotional information is derived from an RGB image (Kinect or webcam). This requires a step involving multimodal data fusion, which is implemented within the Sensor Module (SM), a preprocessor for the Context-Awareness Module.

The architecture was designed to address the limitations of rehabilitation systems that rely mainly on motion-based interaction or therapist-driven parameter adjustment. Four design requirements guided the current implementation: multimodal real-time perception, temporal synchronisation, modular scalability, and rehabilitation-oriented safety. First, the system should integrate heterogeneous sensing modalities, including motion capture, physiological monitoring, wearable sensing, and facial affect estimation, because the user’s interaction state cannot be reliably inferred from a single data source. Second, these modalities should be temporally aligned despite differences in sampling rate, communication protocol, and transmission latency. Third, the system should remain modular and extensible, allowing new sensors, feature extractors, or rehabilitation games to be integrated without redesigning the whole architecture. Finally, therapist-defined constraints and game execution should remain separate from low-level sensor acquisition and fusion in order to preserve rehabilitation-oriented safety. For clarity, the roles of each module are summarised in the following:

- Context-Awareness Module (CAM): Being the core reasoning component, it is designed to interpret all available contextual information such as therapist-defined constraints (received from the web server), gameplay progress (received from the IPM., historical performance (obtained from the database), and, above all, the user’s current physiological and emotional state (obtained from the SM) to generate adaptive recommendations that will be sent to the game(s) and processed by the IPM. As the user’s state depends on multiple input data of different types, the Sensor Module plays an important role by providing higher-level user information to avoid CAM losing time and effort in complex pre-processing tasks.
- Intelligent Play Module (IPM): As a specific game-dependent executing module, its role is to translate CAM-generated recommendations into concrete game-specific actions, such as adjusting task difficulty, repetition count, time constraints, movement amplitude, feedback intensity, exercise order, or session duration while preserving therapeutic objectives and player safety. This separation between CAM and IPM allows high-level reasoning to remain independent of individual game mechanics. The CAM is intended to determine what type of adaptation may be appropriate, while each game-specific IPM determines how such an adaptation should be implemented within a particular rehabilitation game.
- Sensor Module (SM): As a preprocessor for CAM, it acquires heterogeneous sensor data and performs modality-specific preprocessing, temporal alignment, and multimodal fusion to produce a unified representation of the user’s interaction state. This is explained in detail in Section 2.3.

While the CAM and SM are generic proposals; the IPM module is different in every game. Together, these modules define a sensing–reasoning–execution loop. The SM transforms raw multimodal signals into structured user-state representations; the CAM is intended to interpret these representations together with therapist-defined constraints and gameplay feedback, and the IPM is intended to translate CAM directives into game-specific adaptations.

From the perspective of the current development status, the SM has already been implemented and constitutes the main technical focus of this manuscript. In contrast, the CAM is still under design and has not yet been fully implemented. The current IPM prototype operates with fixed-rule-based mechanisms within an adaptive rehabilitation game, and these mechanisms are expected to be replaced or extended by suitable machine learning algorithms in future versions; see [3,5].

### 2.2. Game-Based Testing Scenario

A small study with 43 users was carried out to obtain the first results that could demonstrate the feasibility of multimodal data fusion; see Section 3. The game used for this study is one we are currently developing as a testbed, called Phiby’s Toyland; its architecture and gameplay were recently presented in [5]. Nevertheless, the proposed sensing and fusion framework is not restricted to this specific game. Other rehabilitation-oriented games or interaction scenarios could be connected to the same middleware architecture, provided that they generate compatible motion, physiological, wearable, or affective data streams. Thus, the game can be replaced or modified, while the main sensing, preprocessing, temporal alignment, and fusion mechanisms remain applicable.

**Figure 2:**
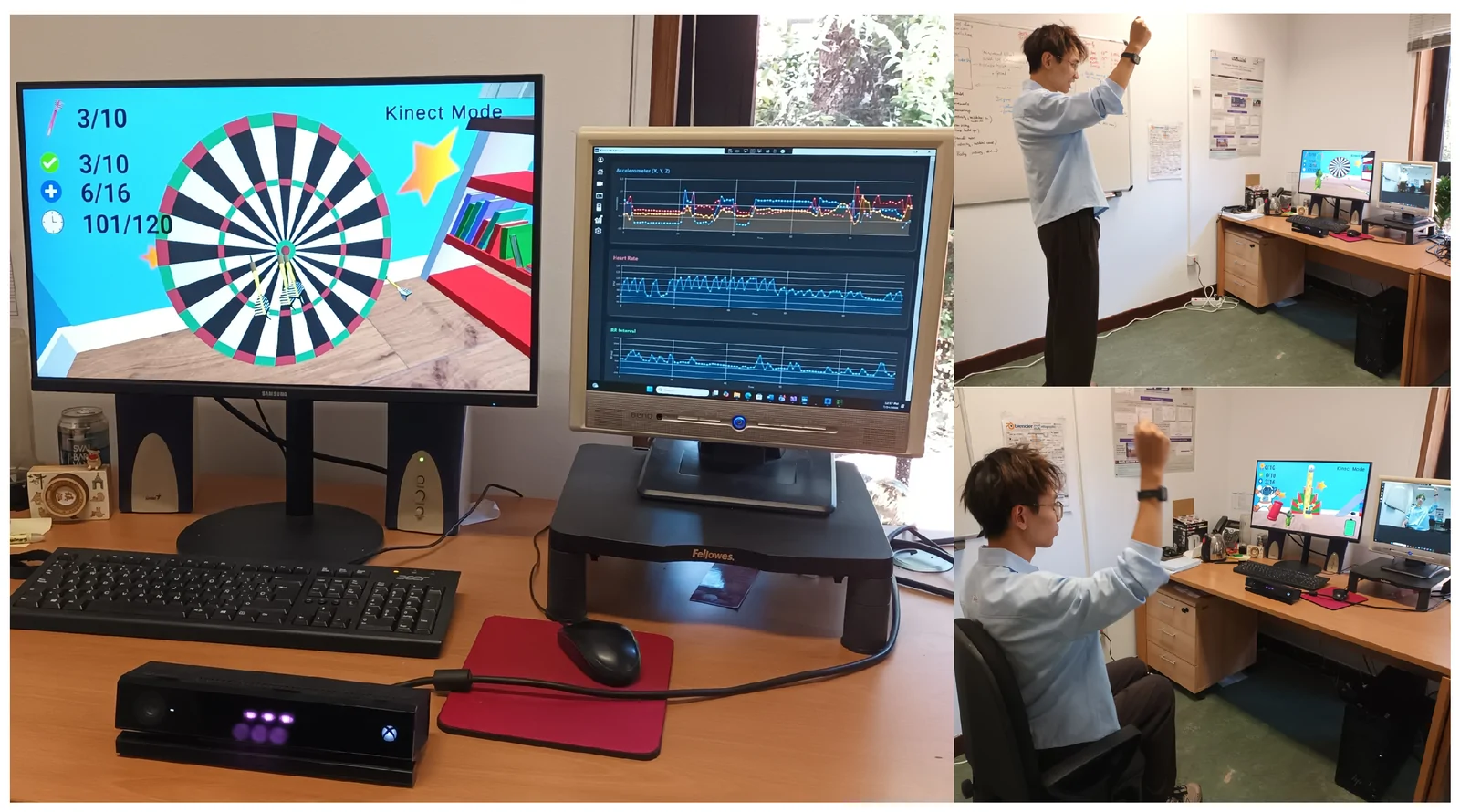
Visualisation of the testing scenario and sensor configuration. (**left**) *Phiby’s Toyland* game screen, Kinect motion capture device, and middleware monitoring interface. (**right**) standing and seated gameplay conditions; the player wears the Bangle.js 2 smartwatch (PUR3 Ltd., Culham, Abingdon, UK) on the right arm and the Polar H10 heart rate sensor (Polar Electro Oy, Kempele, Finland) below their shirt.

The present manuscript does not aim to evaluate the entertainment value, graphical design, interaction design, or therapeutic effectiveness of the game. Instead, it was used to generate multimodal sensor data under real gameplay conditions, allowing the implemented Sensor Module and fusion pipeline to be tested with synchronised motion, physiological, wearable, and affective data streams. To improve the clarity and reproducibility of the experimental scenario, Figure 2 illustrates the game environment and sensor configuration used during data collection. The setup included Phiby’s Toyland gameplay screen, Kinect-based motion capture, RGB-image-based facial affect estimation, and wearable physiological sensors. Participants performed upper-limb movements generally in a standing position or seated if they preferred (the game was suitable for potential wheelchair users), while the sensor streams were synchronised in the middleware for feature-level multimodal fusion.

### 2.3. Sensor Module (SM)

The Sensor Module (SM) was implemented as the acquisition and preprocessing module for the CAM. It collects heterogeneous behavioural, physiological, and affective signals and prepares them for multimodal fusion.

The module currently integrates data acquired from the following three channels: skeletal motion is captured from the Kinect depth camera to obtain information about posture, limb trajectories, and movement execution (details are given in Section 2.3.1). As outlined in Table 1, heart rate data are obtained from two different wearable devices, the Polar H10 chest strap [6] or the Bangle.js programmable smartwatch [7] (Section 2.3.2), and facial affect estimation is derived from facial RGB images, which are processed by a DNN described in Section 2.3.3. These three channels provide the preprocessed data to the following steps of data fusion described in detail in Section 2.4.

#### 2.3.1. Motion Acquisition

The Motion Layer is responsible for capturing and preprocessing full-body movement data and serves as the primary source of behavioural information within the Sensor Module. Motion data are acquired using a markerless motion-capture system based on a Kinect sensor, which provides three-dimensional skeletal tracking in real time [8,9].

The Kinect sensor estimates the spatial positions of the main body joints and segments, enabling continuous monitoring of posture, limb trajectories, and movement amplitude during gameplay. These skeletal data are streamed at a fixed frame rate and time-stamped at the middleware level to ensure synchronisation with physiological and affective modalities.

To support downstream multimodal fusion and context inference, raw skeletal data undergo basic preprocessing, including coordinate normalisation, missing-joint handling, and temporal smoothing. Rather than directly interpreting motion quality at this stage, the Motion Layer focuses on producing stable and time-aligned kinematic descriptors that can be combined with heart rate and emotion signals in later processing stages.

The resulting motion data streams are encapsulated in structured JSON messages and transmitted to the middleware, where they are stored, visualised, and prepared for fusion with other sensor modalities. This design allows motion information to function as a complementary behavioural channel, providing contextual grounding for physiological and affective measurements.

#### 2.3.2. Heart Rate Acquisition

Polar H10 provides reliable electrocardiography (ECG)-based heart rate and RR-interval measurements and has been validated for heart rate variability (HRV)-related analysis [6]. Bangle.js has been added as a more easily wearable device that complements this channel by providing a photoplethysmography (PPG)-based heart rate estimate and accelerometer data. In contrast to commercial smartwatches, direct access to raw sensor streams is possible through an open programmable platform [7].

Using these devices, the SM currently supports heart rate in beats per minute (BPM), RR intervals for HRV-related analysis, tri-axial accelerometer data, affective cues, and signal-quality indicators when available. All incoming streams undergo local preprocessing, including format normalisation, timestamp handling, and basic noise filtering before being passed to the internal fusion component. The processed data are then transmitted as unified JavaScript Object Notation (JSON) packets through the middleware to the Unity (version 2022.3) game engine and stored for real-time visualisation and offline analysis.

For ECG-based monitoring, high-precision heart rate in beats per minute (BPM) and inter-beat interval (IBI) data are captured and parsed by a custom Universal Windows Platform (UWP) application. The processed measurements are formatted into structured data messages and transmitted to the middleware via the Transmission Control Protocol (TCP). Each data packet includes the current BPM value and a sequence of RR intervals with a temporal resolution of approximately ±1 ms, converted from native 1/1024 s units to milliseconds for HRV analysis and visualisation.

In parallel, the PPG branch implemented through Bangle.js provides a lighter and more wearable alternative that is easier to integrate into routine rehabilitation practice. Besides BPM estimation, this channel supplies three-axis accelerometer measurements and a signal confidence indicator, which are useful both for motion-related contextualisation and for assessing the reliability of the optical measurement itself. After local acquisition via the Bluetooth Low Energy (BLE) Nordic UART protocol, the signals are timestamped, normalised, and transmitted to the middleware as structured JSON packets for subsequent fusion.

An additional advantage of this design is extensibility. Although the present implementation focuses on ECG- and PPG-based measurements, the architecture is not restricted to these channels. Further wearable signals, such as gyroscope-derived wrist dynamics or additional physiological indicators, could be incorporated in future versions without altering the higher-level fusion logic.

#### 2.3.3. Emotion Acquisition

To complement motion and physiology with affective cues, the Sensor Module incorporates a facial emotion recognition (FER) submodule we formerly published in [3]. Its purpose is not to provide a psychologically exhaustive interpretation of the user but to deliver low-latency cues related to engagement, valence, and possible tension during gameplay. The emotion sensor performs local real-time emotion inference and forwards its outputs to the preprocessing and fusion component, where affective signals are temporally aligned with motion and physiological data before being delivered to CAM.

The FER pipeline consists of two main stages: face detection and emotion classification. Face detection is performed using a deep neural network (DNN) based on the Single-Shot Detector (SSD) architecture [10] with a ResNet-10 backbone, implemented through OpenCV(version 4.10.0)’s DNN module. The detected face region is then passed to a convolutional neural network (CNN) trained on the FER-2013 dataset [11,12]. To improve robustness and real-time performance in gameplay conditions, the original seven emotion categories were consolidated into four broader affective states: positive, neutral, surprise, and negative. This grouping follows Russell’s circumplex model of affect [13] and allows the downstream fusion layer to focus on general affective trends rather than unstable fine-grained emotion labels.

The CNN was trained using standard regularisation and data augmentation techniques and was later converted to Open Neural Network Exchange (ONNX) format for compatibility with the .NET Framework 4.8. This integration enabled synchronised real-time emotion feedback within the Unity-based BLEXER environment.

### 2.4. Multimodal Data Fusion

This section describes the multimodal feature-level fusion pipeline implemented within the Sensor Module and middleware layer of the BLEXER v3 architecture. The purpose of the pipeline is not just to connect multiple sensing devices, but to transform heterogeneous behavioural, physiological, wearable, and affective data into a common, synchronised, structured, and quality-aware multimodal representation suitable for subsequent context-aware reasoning.

**Figure 3:**
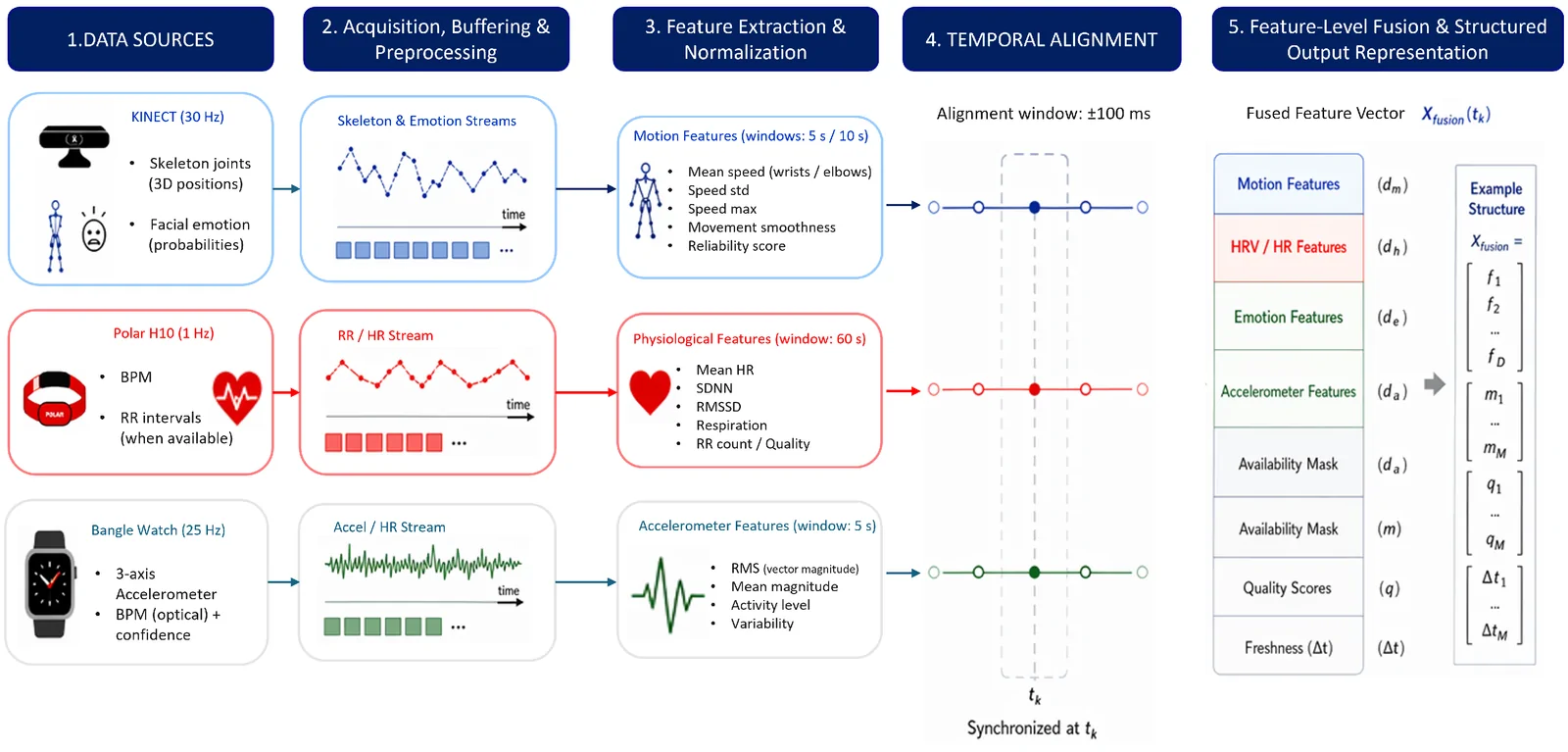
Feature-level multimodal fusion pipeline implemented within the Sensor Module. In the temporal alignment stage, open circles denote sampling points, filled circles denote the observations aligned at $t_{k}$, and the dashed box indicates the ±100 ms alignment window.

The implemented pipeline follows five main processing stages, as illustrated in Figure 3: (1) data sources; (2) acquisition, buffering and preprocessing; (3) feature extraction and normalisation; (4) temporal alignment; and (5) feature-level fusion together with the generation of a structured output representation.

These five stages serve different technical purposes. The data sources provide heterogeneous raw inputs from the Kinect sensor, Polar H10, Bangle.js, and the facial-affect channel. Acquisition, buffering, and preprocessing convert these streams into valid and comparable middleware-level input through timestamp handling, buffering, basic filtering, and signal-quality preparation. Feature extraction and normalisation transform the raw high-frequency signals into window-level descriptors and reduce inter-participant baseline differences through participant-specific normalisation. Temporal alignment maps the modality-specific descriptors to a common reference time $t_{k}$. Finally, feature-level fusion combines the aligned descriptors while preserving modality identity and produces a structured multimodal output representation that also includes metadata describing availability, signal quality, and temporal freshness.

During acquisition, raw data are obtained from Kinect-based skeletal tracking, Polar H10 and Bangle.js physiological sensing, Bangle.js accelerometer sensing, and facial-affect estimation. Each stream is then preprocessed independently through timestamp handling, invalid-sample removal, noise filtering, smoothing, and signal-quality assessment, where applicable. Modality-specific features are subsequently computed within temporal windows and aligned with a common fusion instant. Participant-specific normalisation is applied to selected descriptors, including motion intensity, heart-rate load, and RMSSD, when available. Finally, normalised features are combined with modality-availability indicators, signal-quality metadata, and freshness descriptors to produce the fused output representation $X_{\mathrm{fusion}}\left(t_{k}\right)$.

The output of this process is a fused feature vector, denoted $X_{\mathrm{fusion}}\left(t_{k}\right)$, which combines motion features, heart-rate and HRV-related features, emotion features, accelerometer features, availability information, signal-quality indicators and freshness descriptors. This representation provides CAM with a compact description of the user’s current state, while also preserving information about the reliability and temporal recency of each modality.

#### 2.4.1. Motivation and Design Rationale

In rehabilitation-oriented serious games, the user’s state cannot be reliably characterised through a single sensing modality. Human performance during rehabilitation is multidimensional and involves observable movement execution, physiological regulation, affective response, cognitive engagement, and interaction performance. Consequently, isolated sensor streams may lead to ambiguous or incomplete interpretations of the user’s condition.

For example, elevated heart rate can originate from physical exertion, emotional stress, or positive engagement. Similarly, negative or neutral facial expressions can correspond to frustration, concentration, discomfort, or temporary attentional changes. These examples illustrate that individual modalities provide only partial evidence. A more reliable interpretation requires the joint analysis of heterogeneous information sources, an approach that has also been explored in rehabilitation-oriented serious game systems using wearable and multimodal sensing [14].

For this reason, BLEXER v3 adopts a feature-level fusion strategy. Feature-level fusion was selected instead of early fusion or decision-level fusion because it provides an appropriate compromise between low-level signal preservation and high-level interpretability in the current implementation. This choice is also consistent with multimodal sensing systems in which heterogeneous sensor streams are first transformed into modality-specific descriptors before being combined into a unified representation for downstream interpretation [15,16].

Early fusion, which directly combines raw sensor streams, is not suitable for the present system because the integrated modalities have different sampling rates, temporal resolutions, noise characteristics, and semantic meanings [17]. For example, Kinect skeletal tracking provides high-frequency movement data, heart-rate and HRV-related descriptors evolve on slower physiological timescales, accelerometer data reflect local wearable motion, and facial emotion recognition produces probabilistic affective outputs. Directly concatenating these raw signals would therefore require strong assumptions about synchronisation, scaling, and temporal equivalence that are not appropriate for real-time rehabilitation scenarios.

Decision-level fusion was also not selected at this stage because it would require each modality to provide an independent and validated decision or classifier output before fusion [18]. Such modality-specific classifiers are not yet available or clinically validated in the current proof-of-concept system. In addition, independent single-modality decisions could lead to premature or misleading interpretations, such as elevated heart rate, high intensity of movement, or negative facial affect that may have different meanings depending on the therapeutic task and other available signals.

Feature-level fusion was therefore adopted as an intermediate strategy. Each modality was first transformed into semantically meaningful window-level descriptors. These descriptors were then temporally aligned and combined with availability, signal-quality, and freshness metadata. This design preserved modality-specific information while producing a unified representation that remained interpretable, extensible, and suitable for downstream context-aware reasoning [19,20,21].

#### 2.4.2. Input Modalities and Window-Level Feature Extraction

The fusion pipeline receives input from several sensing channels integrated into the Sensor Module. Each channel contributes complementary information about the rehabilitation process.

The Kinect-based motion stream provides skeletal information at approximately 30 Hz. From the captured joint positions and body posture information, the system derives motion-related descriptors such as mean speed, movement smoothness, speed variation, range of motion, repetition consistency, and tracking reliability. These features describe how the rehabilitation task is physically executed and whether the movement pattern is stable and consistent with the expected exercise.

The Polar H10 chest strap provides ECG-based physiological information, including heart rate and RR intervals. These signals are used to derive heart rate and HRV-related descriptors, such as heart rate, the standard deviation of normal-to-normal (NN) intervals (SDNN), Root Mean Square of Successive Differences between adjacent NN intervals (RMSSD), physiological response related to repetition, and information related to RR count or signal quality when available, following standard HRV measurement principles. These features provide evidence about internal workload, cardiovascular response, fatigue, and autonomic regulation.

The Bangle.js wearable provides a lighter PPG-based heart-rate estimate and tri-axial accelerometer data. The accelerometer stream is used to derive auxiliary movement descriptors such as vector magnitude, root-mean-square (RMS) activity level, mean magnitude, activity variability, and short-term movement intensity. These features can complement Kinect-based motion tracking, especially when skeletal tracking becomes temporarily unreliable due to occlusion or unstable posture estimation. The confidence value reported by Bangle.js is treated as signal-quality metadata rather than as an independent physiological input variable.

The facial emotion recognition pipeline provides affective information extracted from red, green, and blue (RGB) images. Instead of relying on unstable fine-grained emotion classes, the system aggregates the output into broader affective states, such as positive, neutral, surprise, and negative. These affective descriptors provide low-latency cues related to engagement, tension, frustration, or attentional changes during gameplay.

At this stage, gameplay-related information generated by the Intelligent Play Module is not part of the low-level Sensor Module fusion vector. Rather, gameplay feedback is combined with the fused patient-state representation at the CAM level, where sensor-based information, therapist-defined constraints, and game-related feedback can be jointly interpreted for adaptive decision-making.

#### 2.4.3. Feature Computation and Percentile Normalisation

To improve reproducibility, the main feature-computation and normalisation procedures used in the current implementation are summarised in this subsection. Before feature extraction, the incoming sensor streams are converted into a common middleware format containing system timestamps, modality identifiers, signal values, and validity indicators. Invalid or missing samples are explicitly marked and are not forced into the fused representation. Basic preprocessing includes timestamp normalisation, removal of invalid samples, short-term smoothing of motion-derived descriptors, and window-level aggregation prior to fusion.

Motion descriptors are computed from the positions of skeletal joints based on Kinect within each analysis window. Let $p_{j}\left(t_{i}\right)$ denote the three-dimensional position of joint *j* at time $t_{i}$. The frame-to-frame displacement of a selected joint is computed as follows:

(1) $$d_{j}\left(t_{i}\right)={∥p_{j}\left(t_{i}\right)-p_{j}\left(t_{i-1}\right)∥}_{2}.$$

The corresponding frame-level speed is estimated as:

(2) $$v_{j}\left(t_{i}\right)=\frac{d_{j}\left(t_{i}\right)}{t_{i}-t_{i-1}}.$$

Window-level motion intensity is computed as the mean speed across task-relevant joints and frames. Speed variation is computed as the standard deviation of frame-level speed values within the window. Range of motion is estimated as the difference between the maximum and minimum joint positions along the task-relevant movement axis, or as the maximum spatial displacement within the window depending on the exercise type.

Movement smoothness is estimated from the variability of frame-to-frame speed changes; smoother movements correspond to lower variation in speed changes across the analysis window. Repetition consistency is estimated by comparing movement descriptors across detected repetitions of the same exercise, using the variation in duration, amplitude, and movement intensity across repetitions. These motion descriptors are used as operational indicators of movement stability and execution consistency, rather than as clinically validated movement-quality scores.

Heart-rate-related features are computed from Polar H10 heart-rate and RR-interval data. Heart rate is represented in beats per minute (BPM). When a sufficient number of RR intervals is available within the analysis window, HRV-related descriptors are computed, including the standard deviation of NN intervals (SDNN) and the root mean square of successive differences (RMSSD):

(3) $$RMSSD=\sqrt{\frac{1}{N-1}\sum_{i=1}^{N-1}{\left(RR_{i+1}-RR_{i}\right)}^{2}}.$$

These HRV-related features are used as short-term physiological descriptors in the current proof-of-concept implementation and should not be interpreted as clinically validated autonomic markers without longer recordings and clinical validation.

Accelerometer-derived features from the Bangle.js wearable are computed from the tri-axial acceleration signal $\left(a_{x},a_{y},a_{z}\right)$. The acceleration magnitude is calculated as:

(4) $$a_{mag}=\sqrt{a_{x}^{2}+a_{y}^{2}+a_{z}^{2}}.$$

Window-level accelerometer descriptors include mean magnitude, root-mean-square (RMS) activity, activity variability, and short-term movement intensity. The confidence value provided by the wearable is treated as signal-quality metadata.

Facial affective features are obtained from the facial emotion recognition module. For each valid face-detection frame, the module outputs probabilities for the four affective categories used in the current implementation: positive, neutral, surprise, and negative. Window-level affective descriptors include the dominant affective category, the corresponding confidence score, and short-term aggregation of affective probabilities.

Participant-specific percentiles are computed separately for each participant using the valid windows available for that participant. For a feature value *x* and the participant-specific empirical distribution $D_{u}$, the percentile score is computed as:

(5) $$P_{u}\left(x\right)=\frac{\left|\{x_{i}\in D_{u}:x_{i}\leq x\}\right|}{|D_{u}|}\times 100.$$

This transformation is applied to motion intensity, heart-rate load, and, when available, RMSSD. The resulting percentile scores allow each analysis window to be compared with the same participant’s own distribution, reducing the influence of inter-participant differences in baseline movement capacity and cardiovascular response.

#### 2.4.4. Temporal Alignment

A central challenge in multimodal fusion is that the different sensing channels operate at different sampling rates and temporal scales. Skeletal Kinect tracking operates at approximately 30 Hz, Bangle.js accelerometer data are received at approximately 25 Hz, Polar H10 heart-rate updates occur at a lower frequency, HRV descriptors require longer temporal windows, and facial emotion recognition depends on frame-level inference and short-term aggregation. Therefore, strict frame-level synchronisation is neither realistic nor appropriate.

Instead, the system performs timestamp-based temporal alignment on a unified system timeline, since multimodal sensor streams commonly operate at different sampling rates and require window-level aggregation before fusion [15,19]. At each fusion instant $t_{k}$, the pipeline retrieves the most recent valid feature representation from each modality within predefined temporal tolerance windows. Shorter windows are used for motion and accelerometer features, medium windows are used for affective aggregation, and longer windows are used for heart-rate and HRV-related descriptors. This design preserves the temporal characteristics of each modality while still producing a synchronised multimodal representation suitable for downstream interpretation.

Modality-specific feature representations can be expressed as:

(6) $$\begin{array}{cccc} z_{m}\left(t_{k}\right) & =g_{m}\;\left(M_{k}^{\left(w_{m}\right)}\right), & z_{h}\left(t_{k}\right) & =g_{h}\;\left(H_{k}^{\left(w_{h}\right)}\right),\\ z_{e}\left(t_{k}\right) & =g_{e}\;\left(E_{k}^{\left(w_{e}\right)}\right), & z_{acc}\left(t_{k}\right) & =g_{acc}\;\left(A_{k}^{\left(w_{acc}\right)}\right). \end{array}$$

where $z_{m}\left(t_{k}\right)$, $z_{h}\left(t_{k}\right)$, $z_{e}\left(t_{k}\right)$, and $z_{acc}\left(t_{k}\right)$ denote the feature representations of motion, heart-rate/HRV, emotion, and accelerometer data at fusion time $t_{k}$. The functions $g_{m}\left(\cdot \right)$, $g_{h}\left(\cdot \right)$, $g_{e}\left(\cdot \right)$, and $g_{acc}\left(\cdot \right)$ represent the modality-specific feature extraction or aggregation procedures, and $w_{m}$, $w_{h}$, $w_{e}$, and $w_{acc}$ denote the corresponding temporal windows.

#### 2.4.5. Availability and Quality-Aware Representation

Real-world rehabilitation environments can involve temporary signal degradation caused by motion occlusion, packet loss, Bluetooth instability, sensor displacement, illumination changes, or noisy physiological recordings. Therefore, the fusion representation is designed to include not only modality-specific features but also metadata describing the availability, reliability, and temporal freshness of each modality. Including such quality-aware information is important in multimodal healthcare monitoring, where downstream interpretation can be affected by missing, delayed, or unreliable sensor streams [16].

Availability information indicates whether a modality is currently valid, missing, or temporarily unavailable. Signal-quality indicators describe the reliability of the corresponding measurement, such as skeletal tracking reliability, RR interval quality, Bangle.js confidence, or facial emotion recognition confidence. The freshness descriptors represent the time elapsed between the current fusion instant and the most recent valid update of each modality.

The fused feature vector can be expressed as

(7) $$X_{\mathrm{fusion}}\left[t_{k}\right]=\left[d_{m},d_{h},d_{e},d_{a},a,q,\Delta t\right],$$

where $d_{m}$ denotes motion characteristics, $d_{h}$ denotes heart-rate and HRV-related characteristics, $d_{e}$ denotes affective characteristics, $d_{a}$ denotes accelerometer characteristics, *a* represents information on availability of modality, *q* represents indicators of signal-quality and Δt represents the freshness of features descriptors. This structure allows downstream modules to reason not only about the estimated user state but also about the reliability and recency of the information on which that estimation is based.

As a possible future extension, these quality indicators could be used to implement an adaptive weighting or gating mechanism that adjusts the contribution of each modality according to its current reliability. However, this mechanism should be understood as a future development direction, rather than as a fully implemented component of the current system.

#### 2.4.6. Fusion-Derived Operational State Assignment

In addition to generating a quality-aware multimodal feature vector, the fusion layer assigns an operational user-state label for each analysis window using predefined rules. These labels are not intended to represent clinical diagnoses, independently validated user states, or final adaptation decisions. Rather, they provide heuristic and interpretable description of the interaction patterns observed during gameplay, based on the joint behaviour of participant-normalised motion, participant-normalised heart rate, dominant facial affect, and, when available, short-term HRV information.

The current operational state assignment is rule-based and should be interpreted as a proof-of-concept mechanism rather than as an independently validated state-estimation or clinical diagnostic model. To reduce the influence of inter-participant differences in baseline physical capacity, cardiovascular response, and interaction style, motion intensity and heart-rate load are transformed into participant-specific percentile scores. In this representation, a “high” motion or heart-rate value means high relative to the same participant’s own data distribution, rather than high according to a universal clinical threshold. When available, RMSSD is also converted into a participant-specific percentile and used as an auxiliary indicator of short-term autonomic regulation.

The 33% and 67% thresholds were selected as tertile-based operational boundaries to divide each participant’s own distribution into low, intermediate, and high regions. These thresholds were derived heuristically during preliminary technical testing and are not intended to represent clinical benchmarks or medical cut-off values. Similarly, the negative-emotion confidence threshold of 0.40 was used as an engineering threshold to reduce the influence of low-confidence facial emotion outputs when assigning affect-related states. The resulting labels should therefore be understood as operational interaction descriptors within the current proof-of-concept framework. Future work will require therapist annotations, larger datasets, and clinical validation with rehabilitation patients to determine whether these thresholds should be adjusted or replaced by data-driven or clinically informed models. The threshold definitions used in the current implementation are summarised in Table 2.

The rule-based structure also defines how conflicting sensor patterns are handled. In the current implementation, conflicting signals are not treated as errors, and no single modality is allowed to dominate the interpretation automatically. Instead, different combinations of motion, heart-rate load, HRV-related information, and dominant affective cues are mapped to different operational user states. For example, high motion combined with non-negative affect and without elevated heart-rate load or reduced HRV is interpreted as ACTIVE_ENGAGEMENT. In contrast, high motion combined with elevated heart-rate load and either negative affective cues or reduced HRV is interpreted as HIGH_DEMAND. Similarly, elevated heart-rate load without high motion may indicate an affective or cognitive activation pattern rather than a purely physical-load pattern.

When one of the required modalities is missing or invalid, the system does not force a potentially misleading interpretation. Instead, the corresponding analysis window is assigned to the UNCERTAIN category and is treated as a data-availability case rather than as a meaningful user-state estimate. This design allows the fusion layer to preserve ambiguity when sensor evidence is incomplete or inconsistent while still providing transparent operational summaries for downstream context-aware reasoning.

The rule-based assignment logic for the operational user states is summarised in Table 3. The matrix shows how motion level, heart-rate load, emotion, and RMSSD-related conditions were combined to assign each operational state.

- **LOW_DEMAND**: This state is assigned when low motion, low heart rate, and non-negative dominant emotion occur together. It represents a low interaction-demand pattern rather than a clinical condition.
- **MODERATE_STABLE**: This state is assigned to valid multimodal windows that do not satisfy the conditions for LOW_DEMAND, ACTIVE_ENGAGEMENT, PHYSICAL_LOAD, AFFECTIVE_ACTIVATION or HIGH_DEMAND. It represents intermediate or stable interaction conditions.
- **ACTIVE_ENGAGEMENT**: This state is assigned when high motion occurs with non-negative dominant emotion, without concurrent high heart rate or low RMSSD. It suggests active participation without clear evidence of elevated physiological or affective demand.
- **PHYSICAL_LOAD**: This state is assigned when high motion and high heart rate occur together, without dominant negative emotion or low RMSSD. It reflects a pattern of physical-demand mainly associated with the execution of movements.
- **AFFECTIVE_ACTIVATION**: This state is assigned when a high heart rate occurs in the absence of high motion and is accompanied by a negative dominant emotion or a reduced RMSSD. It represents a possible pattern of emotion-related or cognitive activation, rather than a clinical diagnosis of stress or anxiety.
- **HIGH_DEMAND**: This state is assigned when high motion and high heart rate co-occur with negative dominant emotion or low RMSSD. It indicates a higher pattern of interaction-demand that involves physical and affective or autonomic activation.
- **UNCERTAIN**: This category is assigned when at least one required modality is unavailable, such as missing motion information, missing heart-rate information, or unavailable emotion recognition output. It is treated as a data-availability category rather than an interpretable user state.

The resulting labels provide a higher-level summary of the fused multimodal representation. Instead of relying on a single signal, such as motion intensity or heart rate alone, the rule-based assignment mechanism combines physical activity, physiological response, affective cues, and the availability of modality. This allows the fusion layer to assign different operational descriptors to interaction patterns that may appear similar in one modality but differ when interpreted multimodally. For example, high participant-normalised motion may correspond to active engagement when it occurs without elevated heart-rate load or negative affective activation, whereas a similar motion pattern combined with elevated heart-rate load and reduced RMSSD may indicate a higher-demand interaction pattern.

This operational state representation is designed to support downstream context-aware reasoning while avoiding over-interpretation. For example, elevated heart rate alone is not interpreted as excessive workload because it can be associated with physical effort, emotional activation, or positive engagement. Similarly, negative facial affect is not treated as a clinical indicator by itself. Instead, the state labels are derived from multimodal patterns that combine motion, physiological responses, affective cues, and data availability.

#### 2.4.7. Feature Selection and K-Means Clustering Procedure

An exploratory unsupervised clustering analysis was performed to examine recurring data-driven structures within the fused multimodal feature space and to subsequently compare those structures with the predefined rule-based operational descriptors. The analysis was conducted after window-level feature extraction and operational state assignment. However, the operational labels were not used as clustering input variables or optimisation targets.

The input feature vector for each multimodal analysis window consisted of seven fused features: participant-normalised motion percentile, participant-normalised heart-rate load, participant-normalised RMSSD percentile, and four emotion-related probabilities corresponding to positive, neutral, negative, and surprise. Each window was therefore represented as

(8) $$x_{i}=\left[\begin{array}{c} {\mathrm{Motion}}_{i},{\mathrm{HRLoad}}_{i},{\mathrm{RMSSD}}_{i},{\mathrm{EmotionPositive}}_{i},\\ {\mathrm{EmotionNeutral}}_{i},{\mathrm{EmotionNegative}}_{i},{\mathrm{EmotionSurprise}}_{i} \end{array}\right].$$

The UNCERTAIN category was assigned during the rule-based operational state-assignment stage as the default data-availability category. A window was retained for specific operational-state assignment only when participant-normalised motion was available, participant-normalised heart-rate load was available, and the dominant emotion was not unknown. Consequently, windows assigned to UNCERTAIN represented cases in which one or more modalities required for rule-based operational assignment were incomplete or unavailable, including unavailable participant-normalised motion, unavailable participant-normalised heart-rate load, or unknown dominant emotion. These windows were excluded from the exploratory cluster–state comparison.

Windows assigned to MODERATE_STABLE were also excluded from the cluster–state agreement analysis. This descriptor was defined as a broad residual category containing valid windows that did not satisfy the assignment conditions of the more specific operational descriptors. Because it was both broad and dominant in the complete operational-state distribution, its inclusion would have reduced the interpretability of the comparison between data-driven clusters and the more specific rule-based descriptors. This exclusion was specific to the exploratory cluster–state comparison and does not imply that MODERATE_STABLE is invalid or irrelevant to the operational state-assignment mechanism.

After excluding windows assigned to UNCERTAIN and MODERATE_STABLE, windows with missing values in any of the seven clustering features were removed using listwise exclusion. After applying these criteria, 12,675 multimodal analysis windows were retained for the unsupervised analysis.

The retained feature matrix was standardised feature-wise using z-score normalisation:

(9) $$z_{ij}=\frac{x_{ij}-\mu_{j}}{\sigma_{j}},$$

where $x_{ij}$ denotes the value of feature *j* for window *i*, while $\mu_{j}$ and $\sigma_{j}$ denote the mean and standard deviation of feature *j*, respectively, calculated over the retained clustering dataset. This procedure reduced the influence of differences in numerical scale among the selected features.

K-means clustering was then applied to the standardised feature matrix using Euclidean distance. The number of clusters was specified a priori as k=5, corresponding to the number of operational descriptors retained after exclusion of UNCERTAIN and MODERATE_STABLE: LOW_DEMAND, ACTIVE_ENGAGEMENT, PHYSICAL_LOAD, AFFECTIVE_ACTIVATION, and HIGH_DEMAND. This choice was made to support an exploratory comparison between five data-driven groups and the five retained rule-based descriptors, rather than to claim that five represented the optimal number of naturally occurring clusters.

Cluster centres were initialised using the k-means++ strategy. The clustering procedure was repeated using 30 initialisations (n_init = 30), and the solution with the lowest within-cluster sum of squared distances was retained. A fixed random seed (random_state = 529) was used to ensure computational reproducibility.

The rule-based operational labels were not included in the feature matrix and did not influence cluster estimation. After clustering was completed, the pre-existing operational labels were used only to describe the operational-state composition of each cluster. A cluster–state contingency table was constructed by counting the number of windows associated with each operational descriptor within each cluster. Row-wise percentages were then calculated to represent the composition of each cluster.

Overall cluster–descriptor correspondence was summarised using cluster purity:

(10) $$\mathrm{Purity}=\frac{1}{N}\sum_{c=1}^{k}\max_{s}n_{c,s},$$

where *N* is the total number of clustered windows, $n_{c,s}$ is the number of windows in cluster *c* associated with operational descriptor *s*, and $\max_{s}n_{c,s}$ is the number of windows associated with the dominant descriptor within cluster *c*. Purity therefore represents the proportion of clustered windows accounted for by the dominant operational descriptor of each cluster, aggregated across all clusters. It was used only as an exploratory measure of cluster–descriptor correspondence and not as evidence of independent physiological, affective, rehabilitation, or clinical validation.

#### 2.4.8. Interface with the Context-Awareness Module

In our follow-up work, the fused representation $X_{fusion}\left(t_{k}\right)$, together with the corresponding fusion-derived operational user-state label, will be forwarded to the Context-Awareness Module (CAM) to provide it with both numerical multimodal descriptors and compact operational summaries of the current interaction condition.

The fused feature vector preserves modality-specific information, including motion descriptors, heart-rate and HRV-related descriptors, affective cues, accelerometer features, availability indicators, signal-quality metadata, and freshness descriptors. In parallel, the operational user-state label summarises the multimodal pattern observed within the current analysis window. For example, ACTIVE_ENGAGEMENT may support maintaining or gradually increasing task difficulty, whereas HIGH_DEMAND may suggest reducing task intensity, modifying feedback, or introducing a recovery period. UNCERTAIN windows are treated as data-availability cases and should not be interpreted as meaningful user states.

These examples are intended as operational interpretation patterns rather than clinical diagnoses or final implemented CAM rules. In future implementations, CAM can combine these representations with therapist-defined constraints and gameplay feedback, while the IPM translates CAM-generated directives into game-specific adaptations.

## 3. Results

The results presented in this section are based on preliminary laboratory testing sessions designed to evaluate the technical functioning of the implemented multimodal sensing and fusion pipeline. The purpose of these tests was not to assess clinical rehabilitation outcomes but to verify whether heterogeneous sensor streams could be collected, synchronised, transformed into quality-aware multimodal representations, and used to generate operational user-state labels during real gameplay interaction. In addition, the collected data provide an initial real-world gameplay dataset to continue the development of future CAM-oriented machine learning algorithms.

The participants were healthy, non-clinical adult volunteers who tested the prototype system. They were not patients who underwent upper-limb rehabilitation. A total of 43 participants completed the post-game questionnaire and were included in the analysis. The sample included 32 male and 11 female participants. Most of the participants were young adults: 11 participants were between 18 and 20 years old, 28 were between 21 and 25 years old, three were between 26 and 30 years old, and one participant was over 50 years old. Therefore, the dataset should be understood as a preliminary technical validation dataset collected from non-clinical volunteers, rather than as a clinical rehabilitation dataset.

The use of healthy volunteers at this stage was appropriate for testing the stability, synchronisation, and interpretability of the sensing and fusion pipeline before applying the system to more vulnerable rehabilitation users. However, the resulting operational user-state labels should not be generalised to clinical populations. In future work, the system will be retrained and validated with affected people and rehabilitation patients, with therapist annotations and clinical supervision, in order to evaluate whether the proposed fusion-derived representations can support reliable adaptive rehabilitation decisions.

During the sessions, participants played Phiby’s Toyland, a modular serious game for upper-limb rehabilitation developed within the BLEXER framework [5]. The game uses Kinect-based motion capture and includes exercise scenes based on four main upper-body movement categories: alternating arm movement, raising and holding one arm, symmetric arm movements, and trunk movements. In the version used for this data collection, eight exercise scenes were available, including shelf climbing, astronaut flying, darts, toy-house building, balloon inflation, racing, skiing, and a modified Pacman task.

The results presented in this section focus on the output of the implemented multimodal sensing and fusion pipeline. Since the complete decision-making logic of the Context Awareness Module (CAM) is still under development, the present results do not evaluate final adaptive gameplay decisions or clinical rehabilitation outcomes. Instead, they demonstrate how heterogeneous sensor streams collected during real gameplay interactions can be transformed into temporally aligned, quality-aware, and interpretable multimodal representations suitable for downstream context-aware reasoning. The descriptive visual analysis illustrates how the rule-based operational descriptors are distributed within selected dimensions of the fused feature space. In addition, an exploratory K-means analysis examines recurring data-driven structures within the complete fused representation and their correspondence with the predefined operational descriptors. Neither analysis should be interpreted as independent physiological, affective, rehabilitation, or clinical validation of the assigned states.

### 3.1. Multimodal Data Acquisition and Fusion Output

The implemented Sensor Module successfully integrated motion, physiological, wearable, and affective sensing channels within the BLEXER v3 middleware. Motion-related information was obtained from Kinect-based skeletal tracking, physiological data were acquired from Polar H10 and Bangle.js devices, and facial affective information was extracted through the facial emotion recognition pipeline. These streams were received through heterogeneous communication interfaces and converted into structured middleware-level data representations.

After acquisition, each modality was preprocessed and organised into modality-specific descriptors before being temporally aligned on a common system timeline. The resulting fusion output was represented as a structured multimodal feature vector containing motion descriptors, heart-rate and HRV-related features, affective cues, accelerometer information, modality availability indicators, signal-quality metadata, and freshness descriptors. These results demonstrate the technical feasibility of using the implemented Sensor Module to transform asynchronous and heterogeneous sensor streams into a unified, temporally aligned, and quality-aware multimodal representation that can be forwarded to CAM.

### 3.2. Rule-Based Operational State Assignment

The predefined operational state-assignment rules described in Section 2.4.6 were applied to the valid multimodal analysis windows collected during gameplay. Each valid window was assigned an operational descriptor based on participant-normalised motion, participant-normalised heart-rate load, dominant affective information, and, when available, RMSSD-related information.

**Figure 4:**
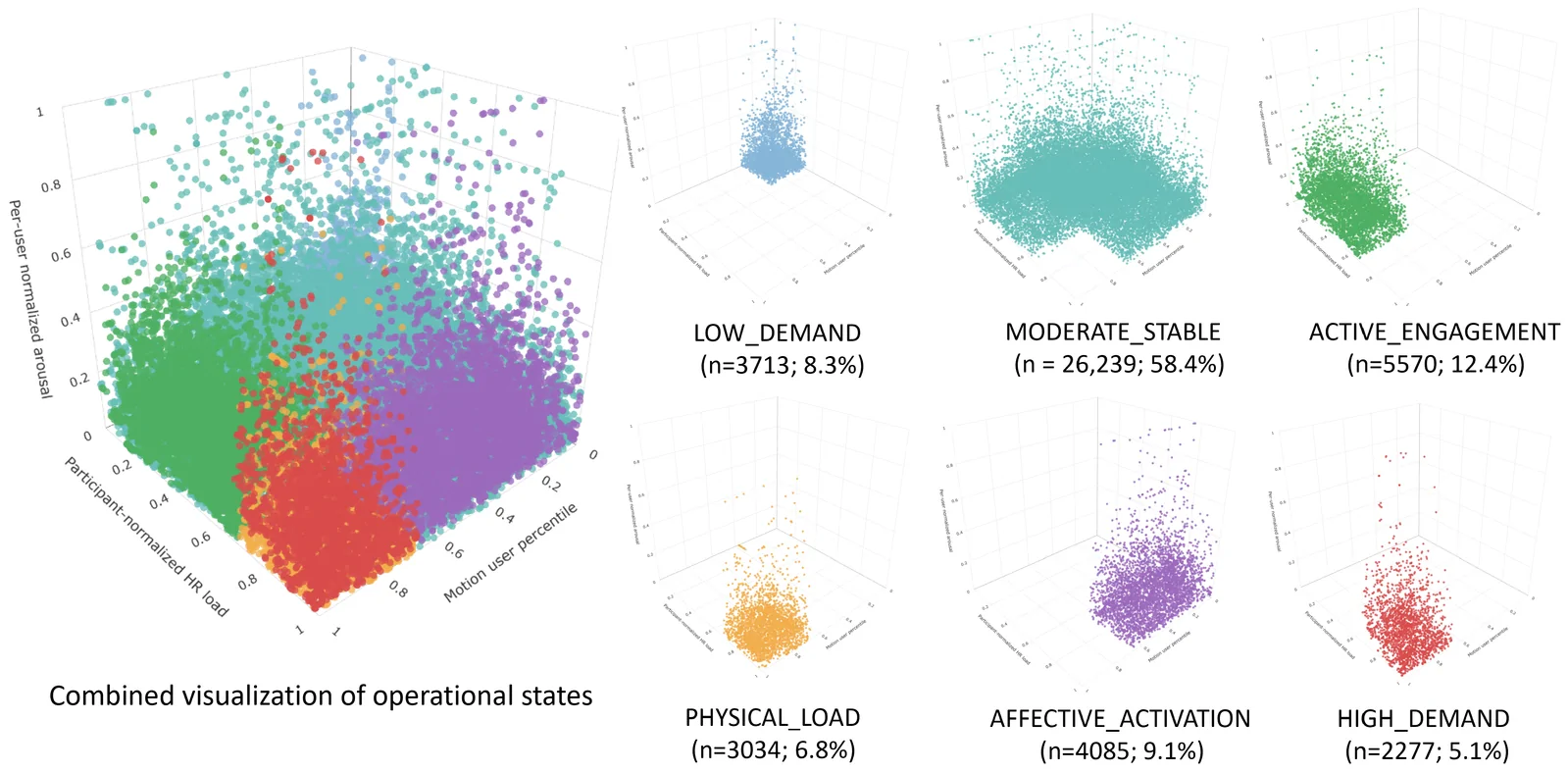
Distribution of the rule-based operational states in the fused multimodal feature space. The combined panel includes all valid analysis windows, while the individual panels show each operational state separately. X = participant-normalised motion percentile; Y = participant-normalised HR load; Z = per-user normalised arousal.

Figure 4 presents the distribution of the rule-based operational state assignments in three selected dimensions of the fused multimodal feature representation. The combined panel shows all valid analysis windows, while the individual panels show the windows associated with each operational descriptor. The displayed axes represent participant-normalised motion percentile, participant-normalised heart-rate load, and participant-normalised arousal.

The operational descriptors occupied partially differentiated but overlapping regions of the selected three-dimensional representation. LOW_DEMAND windows were mainly located in regions characterised by comparatively low motion and heart-rate load. In contrast, PHYSICAL_LOAD and HIGH_DEMAND windows were more frequently observed in regions with higher motion and/or physiological load. AFFECTIVE_ACTIVATION was distributed across a broader range of motion and heart-rate values, reflecting the contribution of affective and RMSSD-related conditions to its rule-based definition.

The MODERATE_STABLE descriptor covered the broadest region of the selected feature space. This distribution is consistent with its definition as a residual category for valid windows that do not satisfy the more specific conditions associated with the remaining operational descriptors. The overlap observed among several descriptors also reflects the fact that the state-assignment mechanism combines multiple features and threshold conditions rather than relying on a single separating variable.

Because the variables displayed in Figure 4 were also used in the predefined state-assignment rules, the observed distributions should be interpreted as a descriptive illustration of the rule-based organisation of the fused feature space. They do not constitute independent validation of the operational descriptors or evidence of classification accuracy.

Windows with incomplete required modalities were assigned to the UNCERTAIN category and were treated as data-availability cases rather than as interpretable operational states. These windows were excluded from the subsequent exploratory cluster analysis.

The MODERATE_STABLE category was also excluded from the cluster–state agreement analysis. As a residual class, it may contain heterogeneous multimodal patterns that do not satisfy the assignment conditions of the other operational descriptors. Including this broad category in the unsupervised comparison would therefore reduce the interpretability of the correspondence between predefined operational descriptors and data-driven clusters.

The remaining operational labels should be understood as heuristic interaction descriptors rather than as clinical diagnoses, independently validated user states, or final adaptation decisions. Their relationship with independently generated data-driven structures was subsequently examined through the exploratory unsupervised cluster analysis presented in Section 3.3.

### 3.3. Exploratory Unsupervised Cluster Analysis

Following the exclusion criteria described above, an exploratory K-means clustering analysis was conducted to examine whether recurring data-driven structures could be identified within the fused multimodal feature space independently of the predefined operational state labels. All participants were pooled, and the analysis included 12,675 valid multimodal windows with complete motion, heart-rate load, RMSSD, and emotion features.

With *k* predefined as five, the K-means procedure partitioned the retained windows into five data-driven clusters, as described in Section 2.4.7. The pre-existing operational labels were not used as clustering input variables or optimisation targets; they were used only after clustering to describe and compare the composition of the resulting clusters. The resulting comparison therefore provides an exploratory assessment of the correspondence between the unsupervised feature-space structure and the predefined rule-based operational descriptors.

**Figure 5:**
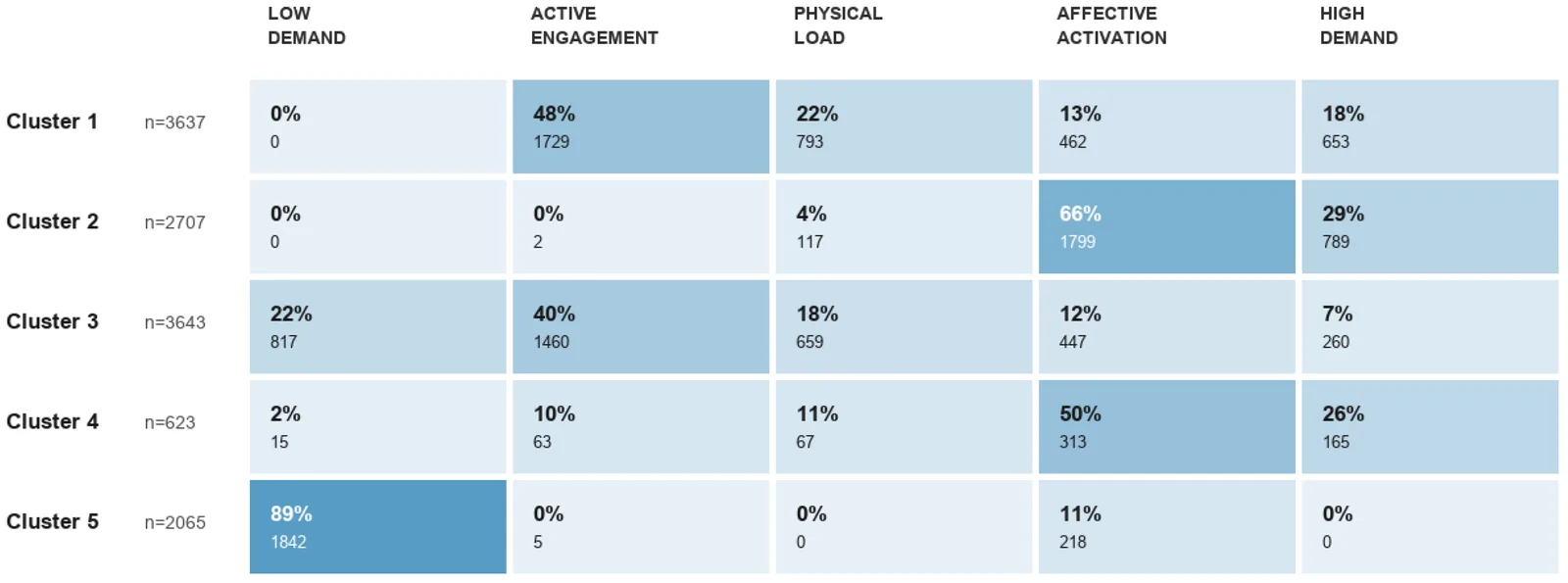
Composition of the global K-means clusters according to the rule-based operational state assignments. Each row represents one cluster and sums to approximately 100%. Percentages indicate the distribution of operational descriptors within each cluster, while the smaller values indicate the corresponding number of multimodal analysis windows. Color intensity reflects the relative proportion within each cluster, with darker shading indicating higher percentages. The MODERATE_STABLE and UNCERTAIN categories were excluded from this exploratory analysis.

Figure 5 presents the composition of each global cluster according to the rule-based operational state assignments. Each row represents one cluster and sums to approximately 100%. The percentages indicate the proportion of each operational descriptor within the corresponding cluster, while the smaller values indicate the number of analysis windows.

The overall cluster purity was 0.564, indicating partial rather than complete correspondence between the unsupervised cluster structure and the predefined operational descriptors. The clusters, therefore, did not show a one-to-one correspondence with the rule-based states. Nevertheless, several clusters showed clear concentrations of particular operational descriptors.

Cluster 5 showed the strongest state-specific concentration. Of the 2065 windows assigned to that cluster, 1842 windows (89%) were labelled LOW_DEMAND, 218 windows (11%) were labelled AFFECTIVE_ACTIVATION, and only five windows (<1%) were labelled ACTIVE_ENGAGEMENT. This indicates that Cluster 5 corresponded to a comparatively concentrated low-demand pattern within the selected fused feature representation. In addition, 68.9% of all LOW_DEMAND windows retained in the analysis were assigned to Cluster 5.

Cluster 2 was dominated by AFFECTIVE_ACTIVATION, which represented 1799 of its 2707 windows (66%). A further 789 windows (29%) were labelled HIGH_DEMAND, while 117 windows (4%) corresponded to PHYSICAL_LOAD. Only two windows were labelled ACTIVE_ENGAGEMENT. The composition of this cluster indicates that AFFECTIVE_ACTIVATION and HIGH_DEMAND shared partially overlapping multimodal feature patterns within the selected representation.

Cluster 4 showed a related but more heterogeneous composition. Of its 623 windows, 313 windows (50%) were labelled AFFECTIVE_ACTIVATION, 165 windows (26%) were labelled HIGH_DEMAND, 67 windows (11%) were labelled PHYSICAL_LOAD, 63 windows (10%) were labelled ACTIVE_ENGAGEMENT, and 15 windows (2%) were labelled LOW_DEMAND. Thus, although AFFECTIVE_ACTIVATION was the dominant descriptor, the cluster also contained several other operational patterns.

Clusters 1 and 3 were more heterogeneous. Cluster 1 contained 3637 windows, of which 1729 windows (48%) were labelled ACTIVE_ENGAGEMENT, 793 windows (22%) were labelled PHYSICAL_LOAD, 653 windows (18%) were labelled HIGH_DEMAND, and 462 windows (13%) were labelled AFFECTIVE_ACTIVATION. No LOW_DEMAND windows were assigned to that cluster.

Cluster 3 contained 3643 windows and was also heterogeneous. The largest proportion corresponded to ACTIVE_ENGAGEMENT, with 1460 windows (40%). This was followed by LOW_DEMAND, with 817 windows (22%); PHYSICAL_LOAD, with 659 windows (18%); AFFECTIVE_ACTIVATION, with 447 windows (12%); and HIGH_DEMAND, with 260 windows (7%).

Overall, LOW_DEMAND showed the clearest cluster-specific concentration, particularly within Cluster 5. In contrast, ACTIVE_ENGAGEMENT and PHYSICAL_LOAD frequently occurred within the same clusters, indicating similarity in their global multimodal feature profiles. Likewise, AFFECTIVE_ACTIVATION and HIGH_DEMAND co-occurred primarily within Clusters 2 and 4, indicating partial overlap between these operational descriptors.

These results suggest that some of the rule-based operational descriptors correspond to comparatively concentrated regions of the fused feature space, whereas others overlap or encompass multiple multimodal patterns. The cluster–state correspondence should therefore be interpreted as exploratory evidence of partial consistency and internal heterogeneity, rather than as independent validation of the operational state assignments.

### 3.4. Implications for Context-Aware Adaptation

**Figure 6:**
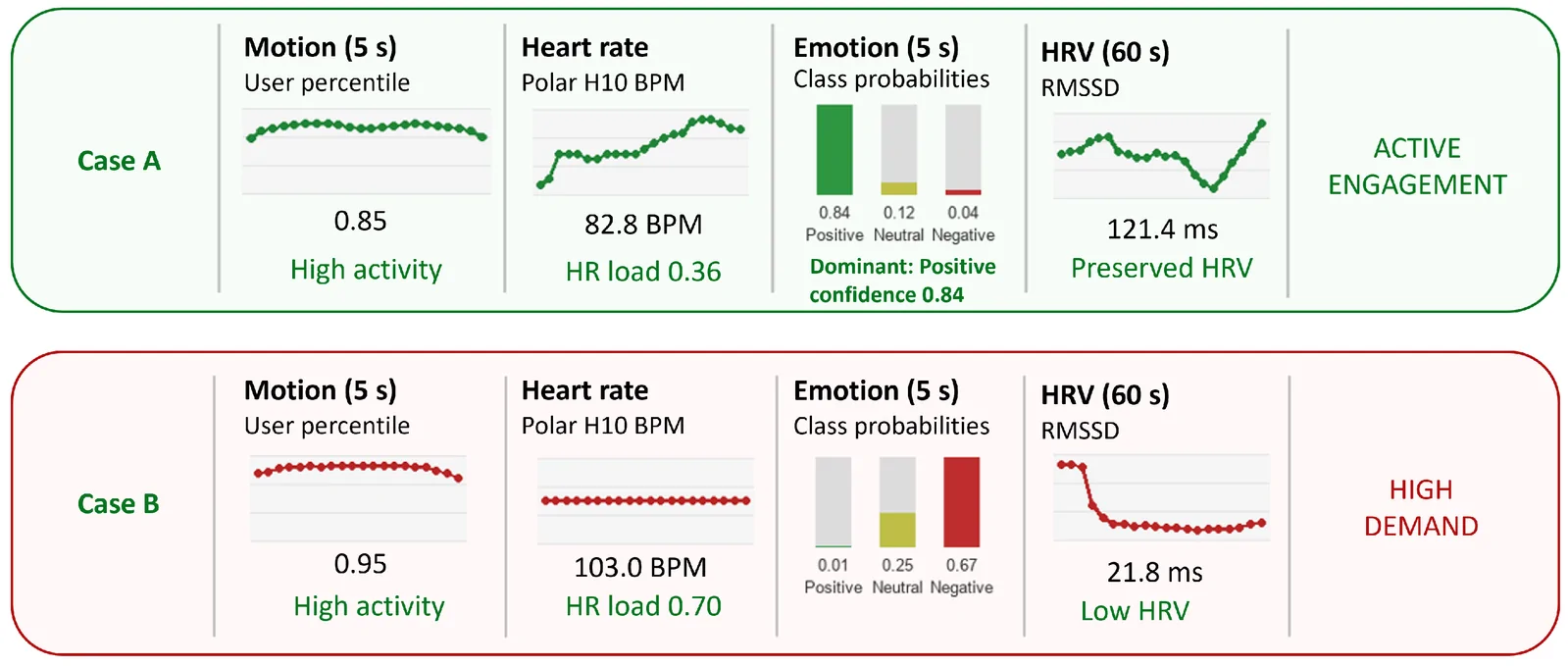
Representative multimodal segments illustrating two fusion-derived operational user states. Case A corresponds to an active engagement pattern, whereas Case B corresponds to a high-demand pattern. These examples are used to illustrate how fused multimodal information can support future CAM-oriented reasoning but should not be interpreted as validated clinical decisions or final implemented CAM rules.

The fusion-derived state representation provides a practical interface between the Sensor Module and CAM. To illustrate how these operational states can support future context-aware reasoning, Figure 6 presents two representative time windows corresponding to different fusion-derived user states.

In Case A, the participant shows high movement activity together with moderate heart-rate load, preserved HRV, and dominant positive affect. This pattern suggests active participation without clear evidence of excessive physiological or affective demand. In contrast, Case B combines high movement activity with elevated heart-rate load, reduced HRV, and dominant negative affective cues, suggesting a higher-demand interaction pattern. These examples show how the fusion-derived representation can help the CAM distinguish between superficially similar high-motion situations that differ in physiological and affective context.

At the current stage, these state labels should be interpreted as operational interaction patterns rather than final adaptive decisions. However, the results show that the implemented fusion pipeline is capable of producing CAM-ready representations that combine physical activity, physiological response, affective state, and data reliability. This provides the technical foundation required for future adaptive gameplay control through the CAM–IPM loop.

## 4. Discussion

This article presented the design and implementation of a multimodal data fusion framework for the BLEXER v3 serious-game ecosystem. The main objective was not to evaluate therapeutic effectiveness or fully autonomous gameplay adaptation but to establish a sensing and fusion layer capable of transforming heterogeneous sensor streams into structured, interpretable, and CAM-ready representations. The results show that the implemented Sensor Module can integrate motion, physiological, wearable, and affective sensing channels and generate quality-aware multimodal feature representations for downstream context-aware reasoning.

The proposed framework extends previous work on the BLEXER ecosystem. Earlier BLEXER versions focused mainly on motion-based interaction, therapist-driven parameter adjustment, and web-based supervision for therapeutic exergames [1,2,4]. Our previous conference paper introduced the conceptual design and global architecture of BLEXER v3 [3], while our previous review work identified user assessment and game control as two foundational components of adaptive serious-game systems [22]. In contrast, the present manuscript focuses specifically on the implemented sensing and feature-level fusion layer, contributing primarily to the user-assessment side rather than claiming to complete the full game-control or autonomous adaptation loop.

From a design-rationale perspective, the choice of feature-level fusion was motivated by the heterogeneous nature of the sensing channels used in BLEXER v3. Motion capture, physiological signals, wearable accelerometer data, and facial affective cues differ in sampling rate, noise characteristics, temporal stability, and semantic meaning. Previous multimodal sensing studies have also shown that heterogeneous sensor streams are often more effectively combined after modality-specific preprocessing and descriptor extraction, rather than directly merging raw signals [15,16,19,20]. Compared with early fusion, the proposed approach avoids direct concatenation of raw signals that are not naturally synchronised or semantically equivalent. Compared with decision-level fusion, it does not require each modality to produce an independent validated decision before integration, which is important at the current proof-of-concept stage.

### 4.1. Assignment and Interpretation of Operational User States

The rule-based operational descriptors provide an interpretable summary layer above the numerical fused feature representation. Rather than interpreting motion, heart-rate load, RMSSD-related information, or facial affect in isolation, the assignment mechanism combines these modalities to distinguish interaction patterns such as low demand, active engagement, physical load, affective activation, and high demand.

The descriptive distribution shown in Figure 4 illustrates how the predefined rules organise the fused feature space. For example, LOW_DEMAND windows were mainly associated with lower participant-normalised motion and heart-rate load, whereas PHYSICAL_LOAD and HIGH_DEMAND were more frequently associated with higher motion and/or physiological load. However, because the displayed variables also contribute directly to the rule-based assignments, this visualisation should not be interpreted as independent validation of the operational descriptors.

The exploratory K-means analysis provides a complementary perspective because the operational labels were not used as clustering input variables or optimisation targets. The overall cluster purity of 0.564 indicates partial rather than complete correspondence between the data-driven cluster structure and the predefined operational descriptors. This suggests that the fused feature space contains recurring patterns related to the rule-based categories, but that the relationship is not characterised by a one-to-one mapping.

The clearest cluster-specific concentration was observed for LOW_DEMAND, which represented 89% of Cluster 5. This suggests that the current rule-based definition of low demand captured a comparatively consistent multimodal pattern within the analysed dataset. In contrast, ACTIVE_ENGAGEMENT and PHYSICAL_LOAD frequently occurred within the same clusters, indicating partial overlap in their global multimodal feature profiles.

Similarly, AFFECTIVE_ACTIVATION and HIGH_DEMAND co-occurred primarily within Clusters 2 and 4. This overlap may reflect shared physiological or affective characteristics, while their rule-based distinction depends on more specific combinations of motion, heart-rate load, RMSSD-related information, and affective cues.

These findings suggest that some operational descriptors correspond to comparatively concentrated multimodal patterns, whereas others encompass overlapping or internally heterogeneous feature structures. The clustering results may therefore support future refinement of heuristic thresholds, subdivision of broad categories, or development of data-driven state-estimation models. However, cluster–descriptor correspondence does not establish physiological, affective, rehabilitation, or clinical validity.

The operational descriptors should therefore continue to be interpreted as task-dependent interaction summaries rather than as universal clinical categories. For example, increased motion intensity and moderate heart-rate elevation may indicate desirable participation during strength- or endurance-oriented exercises, whereas the same response could represent excessive demand during low-intensity or fine-motor tasks. Their final interpretation must remain dependent on the rehabilitation goal, exercise type, therapist-defined constraints, and patient-specific safety requirements.

### 4.2. Importance of the Fusion Step for the Serious-Game Ecosystem

The findings also clarify the role of the present work within the broader BLEXER v3 architecture. The complete CAM decision logic is still under development, and the current IPM prototype remains rule-based. Therefore, this study should be understood as establishing the sensing and fusion foundation for future adaptive game control rather than demonstrating a complete closed-loop autonomous rehabilitation system. Future CAM implementations will need to transform the fused representations and operational state labels into adaptive directives under therapist-defined constraints.

From a software-engineering perspective, the proposed framework was designed to allow the integration of additional sensors and feature extractors without redesigning the entire architecture. A new sensing modality can be incorporated by implementing an acquisition adapter, a timestamping mechanism, a modality-specific feature extractor, and a metadata interface for availability, signal quality, and freshness information. As long as the output follows the common JSON-based middleware representation, the new modality can be added to the fusion vector without modifying the higher-level CAM interface. The replaceable components are mainly acquisition adapters, modality-specific preprocessing functions, feature extractors, and quality-estimation mechanisms, while the fusion interface and CAM-oriented representation remain stable.

From a practical deployment perspective, the framework could support both supervised clinical rehabilitation and future home exercise. In supervised settings, multimodal sensing may help therapists interpret movement execution together with physiological response, affective cues, and signal reliability. In home-based scenarios, the same architecture could provide a basis for remote monitoring and future adaptive gameplay, but the sensor configuration would need to remain simple, low-cost, and minimally intrusive. Additional physiological indicators such as Galvanic Skin Response (GSR) or Electroencephalography (EEG) may provide richer information about arousal, cognitive workload, attention, or stress-related responses, but they may be more suitable for supervised rehabilitation therapy or laboratory validation than for home use. Real-world deployment will therefore require balancing sensing richness with usability, robustness, cost, privacy, and clinical relevance.

### 4.3. Limitations

Several limitations should be considered. First, the current operational state assignment is rule-based and relies on heuristic percentile thresholds. Although participant-specific normalisation helps reduce differences in baseline physical capacity and cardiovascular response, these thresholds require further validation with larger datasets and therapist annotations. Second, the facial emotion recognition component relies on broad affective categories derived from a general-purpose FER dataset. Such models may not fully capture rehabilitation-specific facial expressions, such as concentration, effort, discomfort, or uncertainty. Third, the HRV-related descriptors used in the current implementation should be interpreted with caution. Although a 60 s HRV aggregation window was used for short-term technical testing and real-time interaction analysis, longer aggregation windows may be required to support more robust autonomic regulation analysis, especially in clinical rehabilitation settings. Therefore, the current HRV-related features should be understood as preliminary short-term physiological descriptors rather than clinically validated autonomic indicators.

The exploratory clustering analysis also has methodological limitations. The number of clusters was predefined as k=5 to support comparison with the five retained operational descriptors and was not selected as an estimate of the optimal number of naturally occurring clusters. Consequently, the resulting structure should not be interpreted as evidence that the dataset intrinsically contains exactly five distinct groups.

In addition, K-means clustering relies on Euclidean distance and tends to favour compact distance-based structures. It may therefore provide an incomplete representation if the underlying multimodal patterns are non-spherical, overlapping, or characterised by more complex boundaries. Cluster stability across alternative values of *k*, feature subsets, participant groups, and alternative clustering algorithms was not evaluated in the present study.

The clustering feature vector included four emotion probabilities together with one motion, one heart-rate-load, and one RMSSD-related feature. Although all variables were standardised, the four emotion probabilities form a compositional representation and are not statistically independent. Their inclusion as separate dimensions may therefore have increased the relative influence of the affective modality within the Euclidean feature space.

Finally, cluster purity measures correspondence between the unsupervised clusters and the existing rule-based descriptors. It does not measure physiological, affective, rehabilitation, or clinical validity and should not be interpreted as classification accuracy against independent ground truth.

Finally, the present study should be interpreted as a preliminary technical feasibility evaluation rather than as a complete quantitative validation of the fusion framework. The present results demonstrate the technical feasibility and operation of the implemented fusion pipeline, but they do not establish the effectiveness or superiority of the selected fusion strategy compared to alternative approaches. At this stage, the framework has not been compared with alternative fusion strategies or single-modality baselines, and it has not been evaluated through quantitative state-assignment performance metrics based on independent reference labels, robustness or latency analyses, or expert-labelled ground truth. These evaluations will be addressed in future work once larger datasets, therapist annotations, and clinical user data are available.

## 5. Conclusions

This work presented a multimodal data fusion framework for BLEXER v3, a self-adaptive serious-game ecosystem for upper-limb rehabilitation. The proposed framework integrates Kinect-based motion tracking, Polar H10 and Bangle.js physiological sensing, wearable accelerometer data, and facial affective cues within a middleware-based architecture. The implemented pipeline preprocesses heterogeneous sensor streams, aligns them temporally, and transforms them into quality-aware multimodal feature representations.

In addition to the numerical fusion vector, the framework derives rule-based operational descriptors, including low demand, moderate stability, active participation, physical load, affective activation, high demand, and uncertain. These descriptors are intended to be interpretable summaries of physical, physiological, affective, and data-availability conditions during gameplay rather than as clinical diagnoses or final adaptation decisions.

In addition, an exploratory K-means analysis was conducted using 12,675 complete multimodal windows. With *k* predefined as five, the resulting cluster structure showed partial correspondence with the five retained operational descriptors, with an overall cluster purity of 0.564. LOW_DEMAND showed the clearest cluster-specific concentration, while the remaining descriptors exhibited different degrees of overlap and internal heterogeneity.

These results indicate that the implemented sensing and fusion pipeline can generate structured multimodal representations that contain recurring data-driven patterns. However, the clustering analysis should be interpreted as exploratory evidence of partial correspondence rather than as independent, physiological, affective, rehabilitation, or clinical validation of operational descriptors.

Overall, the proposed framework provides the sensing and feature-level fusion foundation required for future context-aware reasoning through the CAM–IPM loop. Future work will focus on validating and refining operational descriptors using therapist annotations and clinical data, assessing cluster stability and alternative data-driven models, implementing the CAM decision logic, and evaluating the complete adaptive system with rehabilitation users.

## Figures and Tables

**Table 1:** Overview of sensing devices and modalities.

Device/Modality	Raw Data	Extracted Features	Temporal Representation
Kinect/motion	3D skeleton and joint positions	Motion speed, acceleration, variability, and tracking reliability	5 s and 10 s sliding windows with a 1 s step
Polar H10/heart rate	Heart rate and RR intervals	Mean heart rate, SDNN, RMSSD, and valid RR count	60 s rolling window
Bangle.js/heart rate	PPG-based heart rate estimates and triaxial acceleration	Heart rate, confidence, and acceleration-magnitude RMS	5 s sliding window with a 1 s step
Kinect or webcam/ facial affect	Frame-level affective probabilities	Mean affective probabilities, dominant category, confidence, and face-detection rate	5 s sliding window with a minimum 1 s update interval

**Table 2:** Operational threshold definitions used for rule-based operational state assignment.

Level	Rule
Low motion	Motion_user_percentile ≤ 33%
High motion	Motion_user_percentile ≥ 67%
Low heart rate	HR_user_percentile ≤ 33%
High heart rate	HR_user_percentile ≥ 67%
Low RMSSD	RMSSD_user_percentile < 33%
Negative emotion	Emotion_dominant = negative and Emotion_dominant_score ≥ 0.40

**Table 3:** Rule-based operational state assignment matrix. Motion and heart-rate load are divided into low (≤33%), moderate (33–67%), and high (≥67%) ranges.

Operational Descriptor	Data	Motion Percentile			HR-Load Percentile			Emotion		RMSSD	
		Low	Mod.	High	Low	Mod.	High	Non-Neg.	Strong Neg.	Low	Not Low
LOW_DEMAND	✓	✓	–	–	✓	–	–	✓	×	–	–
MODERATE_STABLE	✓	else	else	else	else	else	else	else	else	else	else
ACTIVE_ENGAGEMENT	✓	–	–	✓	✓	✓	×	✓	×	×	✓
PHYSICAL_LOAD	✓	–	–	✓	–	–	✓	–	×	×	✓
AFFECTIVE_ACTIVATION	✓	✓	✓	×	–	–	✓	–	OR	OR	–
HIGH_DEMAND	✓	–	–	✓	–	–	✓	–	OR	OR	–
UNCERTAIN	missing	–	–	–	–	–	–	–	–	–	–

✓: selected rule region; ×: excluded region; OR: either marked condition is sufficient; else: residual rule applied when no more specific state is matched; missing: required modality information is unavailable.

## Data Availability

The data presented in this study are not publicly available because they are part of an ongoing system development and validation process and contain participant-related experimental records. The complete middleware implementation and configuration files are also not publicly available at this stage because they are part of an ongoing development project and include internal system-specific settings. To support reproducibility, the main preprocessing steps, feature definitions, temporal alignment strategy, percentile-normalisation procedure, and rule-based state-assignment logic are described in the manuscript. Further information may be made available from the corresponding author upon reasonable request.

## Abbreviations

The following abbreviations are used in this manuscript:

AI	Artificial Intelligence
BLE	Bluetooth Low Energy
BLEXER	Biosignal and Learning-based EXErcise Regulation
BPM	Beats Per Minute
CAM	Context Awareness Module
CNN	Convolutional Neural Network
DDA	Dynamic Difficulty Adjustment
DNN	Deep Neural Network
ECG	Electrocardiography
EEG	Electroencephalography
FER	Facial Emotion Recognition
GSR	Galvanic Skin Response
HCI	Human–Computer Interaction
HR	Heart Rate
HRV	Heart Rate Variability
IBI	Inter-Beat Interval
IPM	Intelligent Play Module
JSON	JavaScript Object Notation
MLP	Multi-Layer Perceptron
NN	Normal-to-Normal
ONNX	Open Neural Network Exchange
PCG	Procedural Content Generation
PPG	Photoplethysmography
RGB	Red, Green, and Blue
RMSSD	Root Mean Square of Successive Differences
RMS	Root Mean Square
SDNN	Standard Deviation of NN intervals
SG	Serious Game
SM	Sensor Module
SSD	Single-Shot Detector
TCP	Transmission Control Protocol
UWP	Universal Windows Platform
